# GDF15 is an exercise-induced hepatokine regulated by glucagon and insulin in humans

**DOI:** 10.3389/fendo.2022.1037948

**Published:** 2022-12-05

**Authors:** Peter Plomgaard, Jakob S. Hansen, Logan K. Townsend, Anders Gudiksen, Niels H. Secher, Jens O. Clemmesen, Rene K. Støving, Jens P. Goetze, David C. Wright, Henriette Pilegaard

**Affiliations:** ^1^ Department of Clinical Biochemistry, Rigshospitalet, University of Copenhagen, Copenhagen, Denmark; ^2^ Centre of Inflammation and Metabolism and Centre for Physical Activity Research, Rigshospitalet, University Hospital of Copenhagen, Copenhagen, Denmark; ^3^ Department of Clinical Medicine, Faculty of Health and Medical Sciences, University of Copenhagen, Copenhagen, Denmark; ^4^ Department of Human Health and Nutritional Sciences, University of Guelph, Copenhagen, ON, Canada; ^5^ Section for Cell Biology and Physiology, Department of Biology, University of Copenhagen, Copenhagen, Denmark; ^6^ Department of Anaesthesiology, Copenhagen Muscle Research Centre, Rigshospitalet, Copenhagen University Hospital, Copenhagen, Denmark; ^7^ Department of Hepatology, Rigshospitalet, Copenhagen University Hospital, Copenhagen, Denmark; ^8^ Center for Eating Disorders, Elite Research Center for Medical Endocrinology, Odense University Hospital, Odense, Denmark; ^9^ Mental Health Services in the Region of Southern Denmark, Odense, Denmark; ^10^ Clinical Institute, University of Southern Denmark, Odense, Denmark; ^11^ Department of Biomedical Sciences, University of Copenhagen, Copenhagen, Denmark; ^12^ School of kinesiology, Faculty of Land and Food Systems and British Columbia (BC) Children’s Hospital Research Foundation, University of British Columbia, Vancouver, BC, Canada

**Keywords:** fasting, liver, splanchnic bed, appetite, insulin resistance, anorexia nervosa

## Abstract

**Objective:**

Growth differentiation factor (GDF)-15 is implicated in regulation of metabolism and circulating GDF15 increases in response to exercise. The source and regulation of the exercise-induced increase in GDF15 is, however not known.

**Method:**

Plasma GDF15 was measured by ELISA under the following conditions: 1) Arterial-to-hepatic venous differences sampled before, during, and after exercise in healthy male subjects (n=10); 2) exogenous glucagon infusion compared to saline infusion in resting healthy subjects (n=10); 3) an acute exercise bout with and without a pancreatic clamp (n=6); 4) healthy subjects for 36 hours (n=17), and 5) patients with anorexia nervosa (n=25) were compared to healthy age-matched subjects (n=25). Tissue GDF15 mRNA content was determined in mice in response to exhaustive exercise (n=16).

**Results:**

The splanchnic bed released GDF15 to the circulation during exercise and increasing the glucagon-to-insulin ratio in resting humans led to a 2.7-fold (P<0.05) increase in circulating GDF15. Conversely, inhibiting the exercise-induced increase in the glucagon-to-insulin ratio blunted the exercise-induced increase in circulating GDF15. Fasting for 36 hours did not affect circulating GDF15, whereas resting patients with anorexia nervosa displayed elevated plasma concentrations (1.4-fold, P<0.05) compared to controls. In mice, exercise increased GDF15 mRNA contents in liver, muscle, and adipose tissue.

**Conclusion:**

In humans, GDF15 is a “hepatokine” which increases during exercise and is at least in part regulated by the glucagon-to-insulin ratio. Moreover, chronic energy deprivation is associated with elevated plasma GDF15, which supports that GDF15 is implicated in metabolic signalling in humans.

## Introduction

Growth differentiation factor (GDF)-15 was identified in 1997 and classified as macrophage inhibitory cytokine (MIC)-1, suggested to be a TGF-β super family member (1). In 1999, GDF15 was reclassified as a member of the growth differentiation factors and termed GDF15 (2). It was measured in human serum the following year (3). In humans, circulating GDF15 is increased in cancer (4, 5), heart failure (6), obesity (7–9), and also during physiological conditions such as exercise (10) and pregnancy (3). Circulating GDF15 is a biomarker for efficacy of metformin treatment (11) and the weight-reducing effects of metformin is reported to be partly mediated *via* GDF15 in mice (12, 13). However, the metformin-induced increase in GDF15 remains unclear with both the liver (13) and the intestine suggested as the origin (12). Moreover, several cell types express GDF15 and the contribution from various tissues to the circulating GDF15 pool remains to be established.

Several exercise-induced hepatokines have been identified in humans, including fibroblast growth differentiation factor (FGF)-21 (14), follistatin (15), and angiopoietin-like 4 (16), all implicated in energy metabolism and influenced by both fasting and exercise. In mice GDF15 is induced by fasting at the transcriptional level in the liver and as a circulating protein (17). In humans, arterial-to-venous sampling before, during and after a single bout of exercise could not detect a net release of GDF15 from either the resting or the exercising leg (10), suggesting that skeletal muscles do not contribute to the systemic pool of GDF15 and hence other tissues must secrete GDF15. Together, these observations suggest that GDF15 is an exercised-induced hepatokine in humans as previously proposed (18), although this remains unresolved. In addition, glucagon is a stimulus for secretion of hepatokines, both during exercise and fasting, while insulin has an inhibitory effect (14–16). The potential role of glucagon and insulin for regulation of fasting and exercised-induced GDF15 regulation in humans remains unknown. Numerous research groups independently reported that GDF15 was the ligand for the glial cell line-derived neurotrophic factor family receptor alpha like (GRFAL) receptor, which is located in the brain stem and - when activated - results in reduced appetite in rodents (19–22). Furthermore studies in mice suggest that GDF15 can activate the hypothalamic-pituitary-adrenal axis and increase the circulating concentration of glucocorticoid hormones (23).

The aim of this study was to determine whether GDF15 is an exercise-induced hepatokine in humans and examine the relative GDF15 mRNA changes in liver, muscle, and fat by exercise in mice. Furthermore, it was evaluated whether plasma GDF15 is influenced by the nutritional state, and it was also evaluated whether the glucagon-to-insulin ratio influences the regulation of circulating GDF15 in humans.

## Materials and methods

### Human studies

#### Arterial-to-hepatic venous differences

To examine the hepato-splanchnic release of GDF15 during exercise, samples from a previously described study including 10 healthy male subjects (14) were used. In brief, catheters were inserted into the hepatic vein and brachial artery for sampling arterial-to-venous differences over the splanchnic bed. Hepatic blood flow was measured by indocyanine green clearance during a 2-hour cycling exercise at 60% of maximal oxygen uptake (VO_2_max) followed by 4 hours of rest. All subjects reported to the laboratory after an overnight fast and remained fasting during the experimental day.

#### Impact of glucagon-to-insulin ratio at rest

To investigate whether plasma glucagon and insulin regulate circulating GDF15 concentrations, ten healthy males were included in the study. The subjects participated in four separate experimental days in a randomized order. Trial 1) 1 hour of glucagon infusion (GlucaGen, Novo Nordisk) at 6 ng/kg/min. Trial 2) One hour of glucagon infusion at 6 ng/kg/min with co-infusion of somatostatin (Octreotide, Hospira Nordic) at 100 ng/kg/min (started 10 min prior to the glucagon infusion and continued until 120 min). Trial 3) somatostatin infusion at 100 ng/kg/min (same as during trial 2). Trial 4) saline infusion at the same rate as the glucagon infusion rate. At the experimental days, the subjects reported to the laboratory after an overnight fast and were kept resting in a bed and remained fasted throughout the experimental days. Plasma glucagon, insulin and glucose concentrations have been reported (14).

#### Impact of glucagon-to-insulin ratio during exercise

In order to investigate whether the exercise-induced changes in plasma GDF15 depend on the glucagon-to-insulin ratio, samples from a previously performed study were used (24). Six healthy male subjects participated for two experimental days in randomized order. On both days the subjects performed 2 hours of cycling exercise at 60% of VO_2_max and rested for the following 5 hours. On one study day, the subjects exercised under normal conditions and on the other study day, octreotide was infused at 100 ng/kg/min for 2 hours with replacement of glucagon 0.60 ng/kg/min and insulin 0.05 mU/kg/min and glucose was infused to maintain a fasting glucose concentration during the exercise bout. The studies were approved by the local ethical committee (H-1-2012-129) and performed in accordance with the Helsinki Declaration.

#### Healthy male subjects fasting for 36 hours

A total of 17 healthy male subjects participated in the study divided into two groups depending on training status as previously described (25). After a standardized meal at 7 PM, the subjects fasted for 36 hours with free access to water and reported for sampling every 12 hour. Blood samples were obtained 2, 12, 24 and 36 hours after the meal intake. To ensure that the subjects did fast, an increase in ketone body production was monitored (26). HOMA-IR was calculated using plasma glucose and insulin measured after 12 hours of fasting.

#### Patients with anorexia nervosa

Blood was drawn from 25 female patients with severe anorexia nervosa (BMI 13.0 ± 2.0) and 25 age and gender matched control subjects (BMI 21.9 ± 1.4) after an overnight fast. The study groups are presented in reference (27).

### Animal study

Ten-week old male C57Bl6/J mice (Jackson Laboratories) were familiarized to a motorized rodent treadmill (Exer-3R treadmill, Columbus Instruments) for 15 min at 15 m/min and 5% incline on 2 consecutive days (28). Forty-eight hours following acclimation, the mice ran a graded maximal running speed test beginning at 10 m/min at a 5% incline for 3 minutes and increasing speed (3 m/min) every 3 minutes until the mice could no longer maintain the pace for 3 min (28). The maximal performance speed was on average 31.3 m/min. Forty-eight hours following the maximal running test, the mice were randomly selected to remain sedentary or to exercise for 2 hours at 70% of maximal performance speed (21.9 m/min). Mice were anesthetized with sodium pentobarbital immediately post-exercise and tissues were freeze-clamped in situ, quickly removed, and stored at -80°C until analysis. Animal protocols were approved by the University of Guelph Animal Care Committee and followed guidelines established by the Canadian Council on Animal Care.

### Plasma analysis

Plasma GDF15 was measured using a commercially available ELISA assay (R&D systems, human: cat no.: SGD150, and mouse: cat no.: DY6385). Intraplate CV was 2.8% and interplate CV was 5.6%. All samples were analyzed in duplicates. Plasma cortisol and growth hormone were measured using an immunological assays, Elecsys Cortisol II (Roche, Basal, Switzerland) and Elecsys hGH (Roche, Basal, Switzerland) automated on a cobas e801. The assay CV were 8% and 6% respectively.

### Real-time PCR

RNA was extracted using Trizol and Qiagen RNeasy Mini Kits (Qiagen, #74106) and genomic DNA removed using DNase-free treatment (ThermoFisher, #AM1906). cDNA was synthesized using Superscript II (ThermoFisher, #4368814), and real-time PCR was run with SYBR Green Supermix (Bio-Rad, #1725271) using PCR primers on a Bio-Rad CFX Connect system. GDF15 was expressed relative to β-actin using the 2^ΔΔ^Ct method (29). Primer sequences for β-actin were, forward: GACCCAGATCATGTTTGAGA, and reverse: GAGCATAGCCCTCGTAGAT. Sequences for GDF15 were, forward: GAGCTACGGGGTCGCTTC, and reverse: GGGACCCCAATCTCACCT.

### Statistics

Data are presented as mean +/- standard error of the mean (SEM). Differences between groups were evaluated by a two-way repeated-measures ANOVA using a mixed model, where group and time are fixed effects and subjects set as random effect. The *post hoc* analysis was adjusted for multiple comparison using the Tukey test. The statistical analyses were performed in “R” using the lme package and a P value < 0.05 was considered statistically significant. Unpaired two-tailed t-test was used for the animal work.

## Results

### Origin of the exercise-induced increase in GDF15

**Figure 1:**
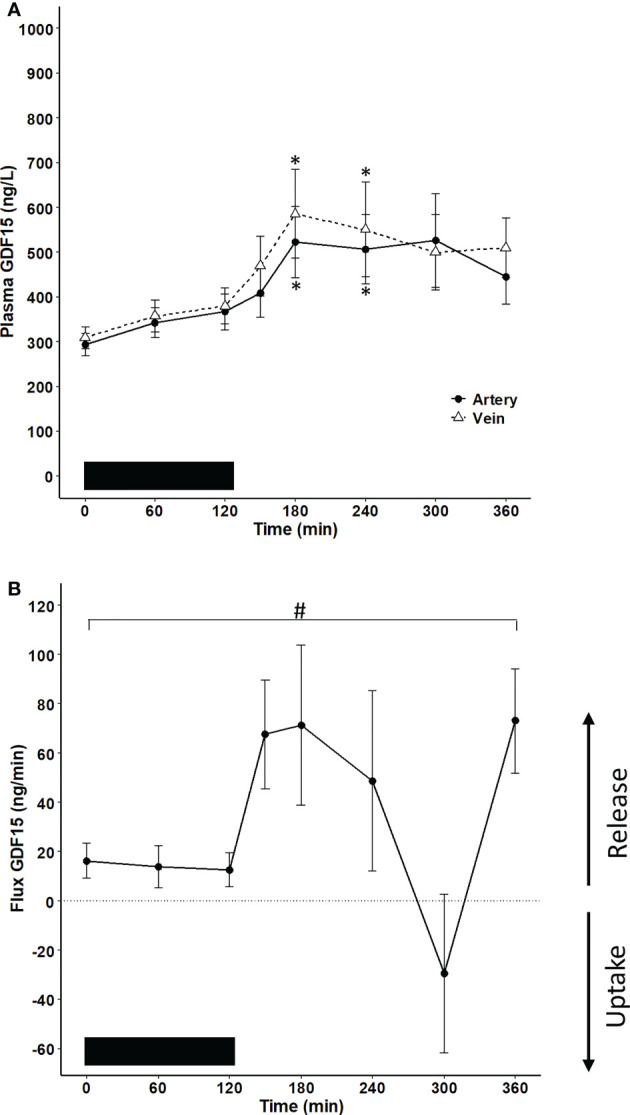
Hepato-splanchnic flux of GDF15 in healthy male subjects (n=10). The black bar represents the exercise bout. **(A)** GDF15 concentration in the artery (black line) and the hepatic vein (broken line) (two-way ANOVA: Time: P <0.0001, Group P=0.1454, TimexGroup=0.9577). **(B)** flux of GDF15 over the hepato-splanchnic bed calculated as the arterial-to-venous difference multiplied by the hepatic flow. A net release from the hepato-splanchnic bed to the circulation as evaluated by the AUC_0 – 360 min_ (P= 0.018). Changes over time were evaluated by a one-way ANOVA (P=0.022). The subjects were performing a cycling exercise from 0 – 120 minutes. Data are presented as means +/- SEM. * significantly different from timepoint “0”, P < 0.05. # AUC is significantly different from zero.

To investigate whether the exercise-induced increase in plasma GDF15 in humans is derived from the hepato-splanchnic bed, the arterio-to-hepatic venous difference for GDF15 was assessed before, during, and after a 2-hour exercise bout. A systemic increase was observed in response to exercise, while the concentration of GDF15 reached a plateau in the recovery after exercise (**Figure 1A**). The plasma concentration of GDF15 was higher in the hepatic vein than in the artery. When the flux over the hepato-splanchnic bed was calculated by multiplying the arterial-to-venous difference of GDF15 with the hepatic plasma flow, a net release of GDF15 from the splanchnic bed was observed throughout the experimental day evaluated by the area under the curve (AUC) (**Figure 1B**). A net release of GDF15 was observed before initiation of exercise, indicating a hepato-splanchnic release of GDF15 after an overnight fast. The hepato-splanchnic release of GDF15 increased with the increase in the arterial GDF15 concentration (**Figures 1A, B**).

### Exercise-induced regulation of GDF15 mRNA in mouse liver, muscle and adipose tissue

**Figure 2:**
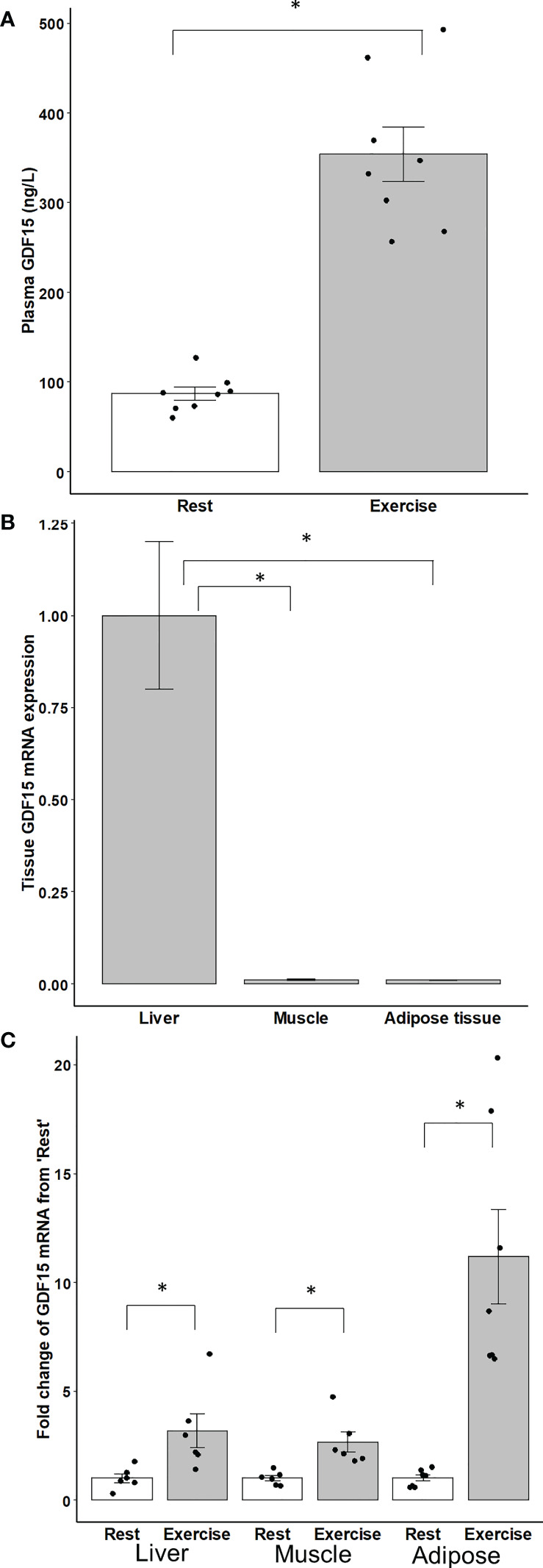
**(A)** Plasma GDF15. **(B)** Content of GDF15 mRNA in liver, muscle and adipose tissue. **(C)** Fold change from ‘Rest’ of GDF15 mRNA in liver, skeletal muscle (triceps), and adipose (epididymal) tissue in male mice immediately after 2 hours of running exercise (n=8) compared to the rest group (n=8). Data are presented as means +/- SEM. *: P < 0.05.

Tissue expression of GDF15 was examined in mice performing an acute bout of running exercise. Circulating GDF15 concentrations were ~3-fold higher immediately post-exercise compared to resting mice (**Figure 2A**). To assess the potential tissue source of increased circulating GDF15, GDF15 mRNA was determined in liver, epididymal white adipose tissue, and triceps muscle. Liver GDF15 mRNA content was 98-fold and 88-fold higher than adipose tissue and muscle (**Figure 2B**). However, GDF15 mRNA increased with exercise in all examined tissues (**Figure 2C**).

### Influence of glucagon and insulin on circulating GDF15 in humans

**Figure 3:**
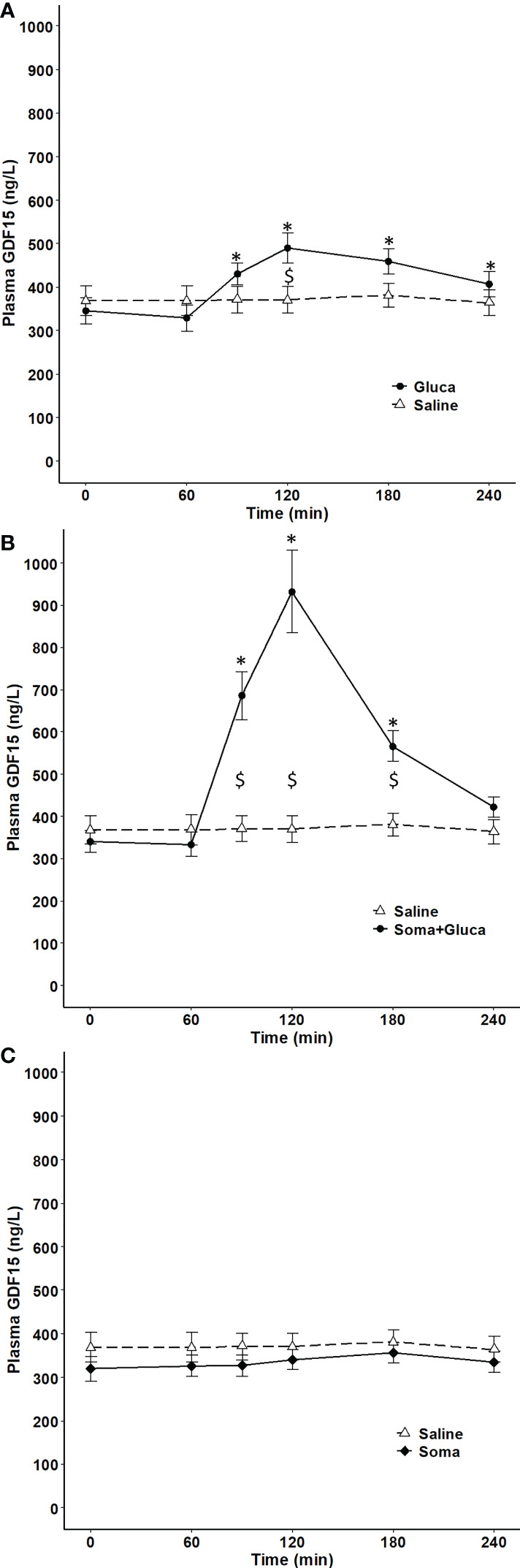
Plasma GDF15 in 10 healthy males completing 4 separate trials. On one day, the subjects received 1 hour of saline infusion (control condition) (broken line in **A-C**). On other days, the subjects received a) Condition with elevated glucagon and insulin by 1 hour of glucagon infusion (0 – 60 min) (two-way ANOVA: Time P <0.0001, Group P= 0.3512, TimexGroup P< 0.0001) **(B)** Condition with elevated glucagon and reduced insulin by 1 hour of glucagon infusion (0 – 60 min) + somatostatin (-10 - 120 min) (two-way ANOVA: Time P < 0.0001, Group P= 0.0023, TimexGroup P< 0.0001) and **(C)** Condition with suppression of both glucagon and insulin by infusion of somatostatin (-10 – 120 min) (two-way ANOVA Time P=0.0562 Group P=0.3403 TimexGroup P=0.5728). Data are presented as means +/- SEM. * significantly different from timepoint “0” and “$” indicates a difference between groups P< 0.05). Plasma concentrations of insulin, glucagon and glucose are published in Figure 4 in reference (14).

**Figure 4:**
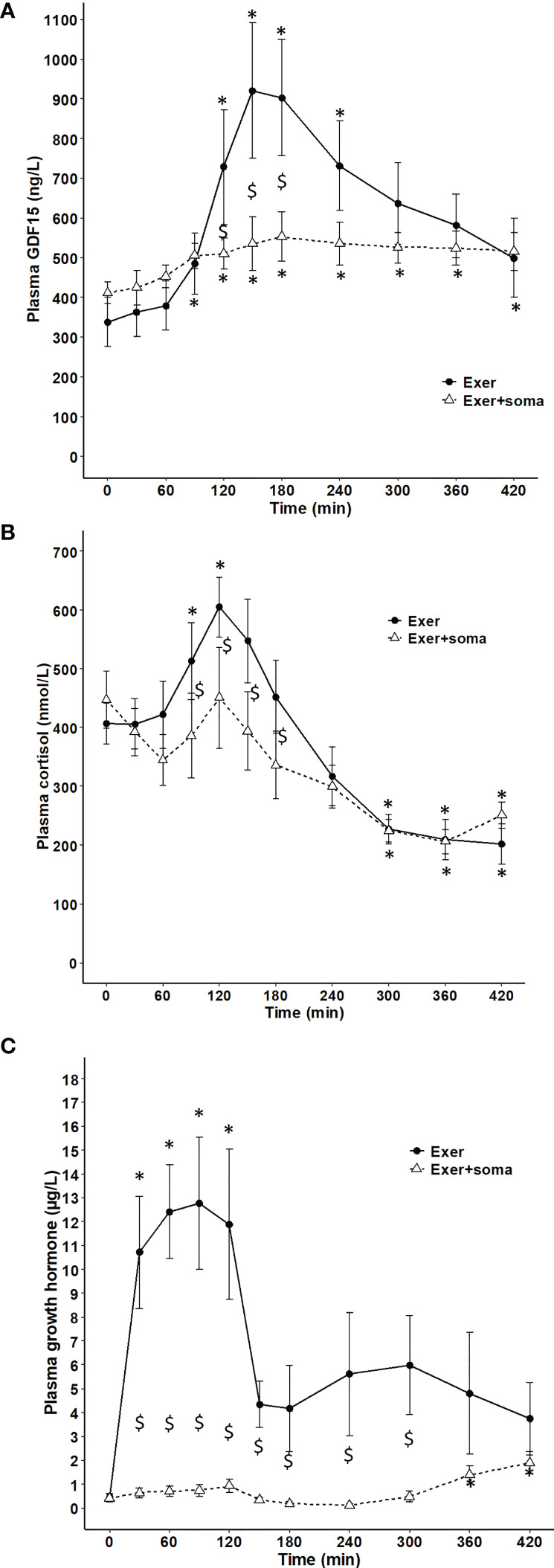
Plasma GDF15 and cortisol in 6 healthy males performing 2 hours of cycling exercise on 2 separate days with no infusion (black line) or with infusion of somatostatin (broken line). **(A)** plasma GDF15 (two-way ANOVA: Time P< 0.0001, Group P=0.0013, TimexGroup P= 0.0044). **(B)** plasma cortisol (two-way ANOVA: Time P< 0.0001, Group P=0.0028, TimexGroup P= 0.0877). **(C)** plasma growth hormones (two-way ANOVA: Time P< 0.0001, Group P< 0.0001, TimexGroup P< 0.0001). Data are presented as means +/- SEM. * significantly different from timepoint “0”, P < 0.05 and “$” significantly different from control, P < 0.05. Plasma concentrations of insulin, glucagon and glucose are published in reference (24).

The dependence of circulating GDF15 on the glucose regulatory hormones glucagon and insulin was investigated in resting subjects. One-hour infusion of glucagon resulted in a plasma concentration corresponding to that observed during exercise and associated with a modest (~1.4-fold) increase in circulating GDF15 (**Figure 3A**). However, as insulin increases during glucagon infusion and counter-acts the effects of glucagon, the 1-hour glucagon infusion was repeated on a separate day with co-infusion of somatostatin (to block insulin) and resulted in ~3-fold increase in the GDF15 concentration (**Figure 3B**). Infusion of saline or somatostatin alone did not result in any change in the GDF15 concentration (**Figure 3C**). Moreover, blunting the increase in glucagon-to-insulin ratio during exercise by a pancreatic clamp using somatostatin was associated with complete prevention of the exercise-induced increase of plasma GDF15 (**Figure 4A**). The exercise-induced increase in cortisol (120 min) preceded the peak in exercise-induced GDF15 (150 – 180 min), (**Figure 4A** compared to **4B**). Somatostatin blunts the exercise-induced increase in cortisol, (**Figure 4B**). Growth hormone is increased during the 2-hour exercise bout and, this is blocked by the somatostatin infusion, (**Figure 4C**).

### Influence of reduced nutritional state on plasma GDF15

**Figure 5:**
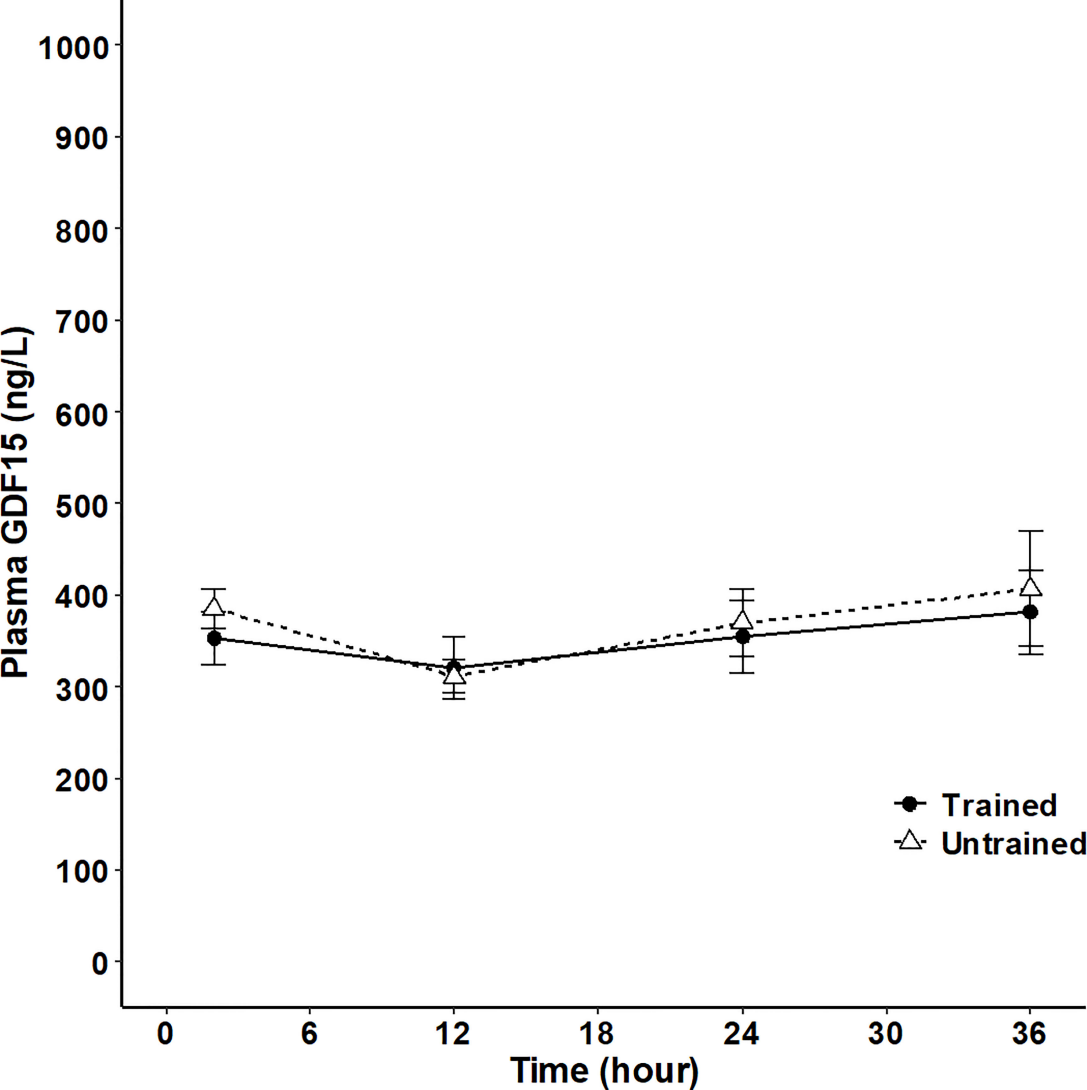
Plasma GDF15 in 8 trained (black line) and 9 untrained (broken line) healthy male subjects at 2 hours, 12 hours, 24 hours and 36 hours after a standardized meal. (Two-way ANOVA: Time P<0.0377, Group P=0.7864, TimexGroup P= 0.9320). Data are presented as means +/- SEM.

**Figure 6:**
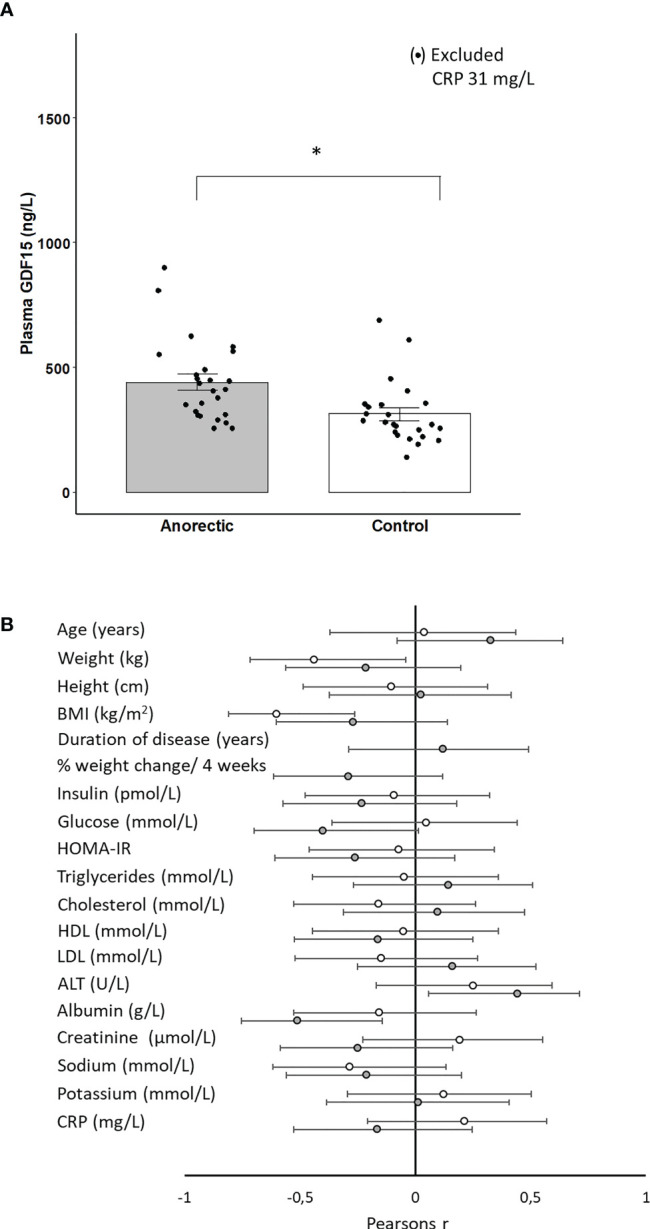
**(A)** Plasma GDF15 in patients with anorexia (n=25) and matched controls (n=25). In the control group, one measurement was excluded from the mean as the subject had elevated CRP. * Significant difference between groups P < 0.05. **(B)** Plasma GDF15 was correlated to the basal characteristics of the anorectic patients (n=25) (gray circles) and controls (n=24) (open circles). BMI: Body Mass Index, HOMA-IR: Homeostatic Model Assessment for Insulin Resistance, ALT: alanine aminotransferase, CRP: C-reactive protein. The figure depicts Pearsons r with 95% confidence intervals.

Finally, GDF15 was assessed both during short-term (36 hours) fasting and in patients with anorexia nervosa. The fasting period of 36 hours did not result in a significant change in circulating GDF15 and there was no difference between the trained and untrained group (**Figure 5**). However, plasma GDF15 was 1.4-fold higher in patients with anorexia nervosa than in age-matched healthy subjects (**Figure 6A**). Plasma GDF15 correlated to body weight and BMI in the control group and in patients with anorexia nervosa. Moreover plasma GDF15 correlated negatively to plasma albumin and positively to alanine aminotransferase (ALT) (**Figure 6B**).

## Discussion

The present study establishes that, in humans, the hepato-splanchnic bed contributes to the circulating GDF15 pool. The study furthermore demonstrates that the GDF15 secretion is stimulated by glucagon and inhibited by insulin during rest. In addition, the exercise-induced increase in circulating GDF15 is blunted by a pancreatic clamp, a condition where glucagon and insulin levels remain unchanged during exercise. Taken together, these data demonstrate that exercise-induced increase in plasma GDF15 is at least in part regulated by the glucagon-to-insulin ratio in humans, and that the liver is the most likely source with an increased GDF15 release from the hepato-splanchnic bed and increased hepatic GDF15 transcription in mice.

Several tissues affected by exercise express GDF15, including the working skeletal muscles, adipose tissue, liver, and the heart. Cultured muscle cells exposed to electrical pulse stimulation secrete GDF15 to the medium (30), and despite a low expression in the skeletal muscles, exercise induces a transient increase in muscle GDF15 mRNA in mice (31). Both findings suggest that GDF15 could be a myokine. In humans, however, while plasma GDF15 increases in response to exercise, no release from the exercising nor from the resting leg can be detected when measuring the artery-to-venous difference (10). Thus, human leg tissues do not contribute to the circulating GDF15 concentration, suggesting that if the contracting muscle cells do secrete GDF15 in humans, it has no endocrine role. Of note, no transcriptional change of GDF15 mRNA was observed in muscle biopsies from healthy subjects or patients with type 2 diabetes in response to exercise (32). However, GDF15 could act locally within the leg, but an autocrine effect seems unlikely because the muscle cell does not express the GFRAL receptor, at least not in mice (19). On the other hand, a paracrine effect on the adipose tissue is possible as the human adipose tissue express mRNA for the GFRAL receptor (30). The present findings confirm that skeletal muscle and adipose tissue from mice have a much lower mRNA level of GDF15 than in the liver as the liver from mice has the highest resting level of GDF15 mRNA (33) which increases in response to “stress” induced by e.g. ethanol, chloroform, and partial hepatectomy (33). The present observation that acute exercise markedly increases the GDF15 mRNA content in the liver provides evidence that hepatic GDF15 is also regulated by a mild physiologic stress. Because GDF15 mRNA increases in liver, muscle, and adipose tissue in response to exercise in mice, it is difficult to determine the relative contribution to the circulating GDF15 concentration but the hepato-splanchnic release of GDF15 suggests that exercise-induced GDF15 in the circulation is derived mainly from the liver. In the present study an uptake of GDF15 from the hepato-splanchnic bed, was observed in the recovery after exercise (timepoint 300 min), however it cannot be ruled out as an artifact. Whether the adipose tissue and the heart also contribute to the exercise-induced increase in circulating GDF15 concentrations remains unknown.

The primary target of circulating glucagon is the liver, and the present finding that exogenous glucagon increased the circulating concentration of GDF15 supports that the liver is also the source of exercise-induced GDF15 in humans. Both glucagon and insulin act on hepatocytes. When only administering glucagon (**Figure 3A**), plasma glucose increases and thereby insulin (14) and a modest increase in GDF15 was observed which demonstrates that insulin has an inhibitory effect on GDF15 secretion. Thus, the ratio between glucagon and insulin contributes to regulating hepatic GDF15 secretion during exercise. Glucagon also stimulates the secretion of other hepatokines, including FGF21 (14), angiopoietin-like-4 (16), and follistatin (15) in the absence of insulin or when insulin is only present in low concentrations as during an exercise bout. The secretion of these hepatokines has been confirmed *in vitro* where the signal mediated by glucagon is transferred by cAMP as second messenger in the hepatocyte (14–16). Transcription of GDF15 is regulated by the cAMP response element binding protein 1 (CREB1) in a human pancreatic cancer cell line (34), making cAMP a likely second messenger also for exercise-induced GDF15 production in the liver. Biguanides such as metformin increase the hepatic GDF15 expression in mice (13) and in the circulation in humans (11). In contrast, at least in mice, metformin seems to act *via* a glucagon antagonistic mechanism by reducing the cAMP levels in the hepatocyte (35). However, others have demonstrated metformin to increase hepatic GDF15 mRNA and secretion *via* activating transcription factor 4 (AFT4) and CCAAT-enhancer-binding protein homologous protein (CHOP) involved in cell stress (13, 31), a signaling pathway also found in a mouse model of non-alcoholic steatohepatitis to increase the GDF15 mRNA level in the liver (36). Hepatic ER stress increases in mice after exercise (37, 38), but a recent study in mice demonstrated that during exercise, also in mice, GDF15 secretion occurs independent of ER stress/CHOP (39). In humans, *in vivo*, regulation of plasma GDF15 by the glucagon to insulin ratio is compatible to other physiological and pathophysiological conditions. Elevated plasma GDF15 is observed in patients with impaired insulin sensitivity (9) and plasma GDF15 concentration is independently associated with insulin resistance measured by HOMA-IR (40). As insulin has an inhibitory effect on GDF15 secretion, hepatic insulin resistance may augment secretion of GDF15. Thus, the proposed regulatory mechanism by the glucagon-to-insulin ratio during exercise seems relevant for conditions associated with impaired insulin sensitivity.

In mice, hepatic GDF15 mRNA increases, as well as circulating GDF15 increase after only a fasting period of 24 hours (17). In humans a transient increase in plasma GDF15 with a peak at 48 hours has been reported for a 7-day fasting period (41). We could not detect an effect on plasma GDF15 during a 36-hour fasting period and there was no difference between the trained insulin sensitive group compared to the untrained insulin resistant group HOMA-IR (1.44 ± 0.3 v 2.81 ± 0.5 P= 0.046) (25). The fasting period for plasma FGF21 to increase in mice is 24 hour (42) and in humans 7 – 10 days (43, 44). Thus, whether a period longer than 7 days of fasting in humans increase plasma GDF15 remains unknown.

The present study demonstrates that patients with anorexia nervosa have higher plasma GDF15 than matched controls. GDF15 correlated with low albumin and high levels of alanine aminotransferase in the anorectic group but not in the control group, which supports that GDF15 in patients with anorexia nervosa may be liver-derived. In addition, both low albumin and elevated alanine aminotransferase are markers for evaluating liver function, indicating that hepatic “stress” mediates an increase in plasma GDF15 in patients with anorexia nervosa.

In the present studies somatostatin was used to manipulate the glucagon-to-insulin ratio in healthy subjects. Somatostatin also blocks the secretion of cortisol and growth hormone. In patients with cortisol deficiency GDF15 is elevated (45), however when resting healthy subjects are infused with somatostatin no change is observed in plasma GDF15. Of note, GDF15 is suggested to mediate the inflammatory-induced increase in cortisol (23), but from the kinetic patterns of the curves the exercise-induced cortisol peak precedes (120 min) the peak in GDF15 (150 -180 min) **Figures 4A, B**, suggesting a minor role for GDF15 in cortisol regulation during an acute exercise bout. Plasma growth hormone is elevated during exercise and blocked by the infusion of somatostatin during exercise. The exercise-induced increase in growth hormone could contribute to the stimulation of GDF15, however this seems unlikely as patients with growth hormone deficiency have elevated levels of GDF15 (46).

Interest in the function of GDF15 as an endocrine signal has centered around appetite regulation because GDF15 is the ligand for the GFRAL receptor, and GDF15 reduces appetite in mice (19–22). We and others (10) observe that exercise-induced GDF15 is increased both during and in the hours after exercise when the body needs to replenish its energy storages. This makes it difficult to reconcile GDF15 as a pure appetite reducing hormone during normal physiological conditions (47). Besides exercise, a physiological increase of plasma GDF15 is observed during pregnancy (3, 48), which also seems contradictory with a suppression in appetite, in the light of the increased energy requirements during development and growth of the fetus. Interestingly at least in mice, physiologically elevated GDF15 by exercise does not inhibit appetite or suppress wheel running activity whereas pharmacologically elevated GDF15 in mice does (49). This suggests that the role of physiological elevated GDF15 in human physiology is unclear and has yet to be identified. In pathological conditions, such as cancer and heart failure, associated with cachexia, an elevated circulating concentration of GDF15 is easier to reconcile with a reduction in appetite and food intake, but it seems counter-intuitive that patients with anorexia nervosa tormented by chronic hunger demonstrate elevated GDF15. The patients with anorexia nervosa are characterized by low levels of the satiety hormone leptin (50), whereas the hunger hormone ghrelin is elevated (51). Thus, a low plasma concentration of GDF15 would be expected in patients with anorexia, rather than an elevated level (**Figure 6A**). Of note it could be speculated the elevated GDF15 concentration observed in patients with anorexia nervosa could be a part of the pathology and not secondary to the energy deprivation. This would point to antagonizing GDF15 as a therapeutic target for treatment of anorexia nervosa. However, the function of circulating GDF15 remains to be solved in humans.

## Conclusion

The present results demonstrate that exercise induces a hepato-splanchnic release of GDF15 to the circulation and that the release depends on the glucagon-to-insulin ratio in humans. In support, GDF15 mRNA increases in mouse liver in response to exercise. Taken together, these findings provide evidence that GDF15 is a hepatokine and related to energy metabolism. However, of relevance not only to exercise physiology but also for evaluation of pathophysiological conditions associated with insulin resistance, the endocrine effects of GDF15 need to be unraveled.

## Data availability statement

The raw data supporting the conclusions of this article will be made available by the authors, without undue reservation.

## Ethics statement

The studies involving human participants were reviewed and approved by De Videnskabsetiske Komiteer - Region Hovedstaden. The patients/participants provided their written informed consent to participate in this study. The animal study was reviewed and approved by University of Guelph Animal Care Comittee.

## Author contributions

PP, JH, AG, NS, JC, RS, JG, and HP performed the human studies, laboratory analysis and data analysis and interpretation. LT and DW performed the animal studies, laboratory analysis and data analysis and interpretation. All authors critically revised the manuscript and read and approved the final version of the manuscript.

## Funding

The Centre for Physical Activity Research (CFAS) is supported by TrygFonden (grants ID 101390 and ID 20045). During the study period, the Centre of Inflammation and Metabolism (CIM) was supported by a grant from the Danish National Research Foundation (DNRF55). This study was further supported by a grant from the Augustinus Foundation. LT was supported by the Natural Sciences and Engineering Research Council of Canada. DW is a Tier II Canada Research Chair. Animal work was supported by the Discovery Grant from the Natural Sciences and Engineering Research Council of Canada. Danish Ministry of Culture for Sports Research and Danish Council for Independent Research.

## Acknowledgments

We thank all study participants for their cooperation in this project. The authors are grateful for the excellent technical support provided by lab technician Anne Truesen Asanovski from the Department of Clinical Biochemistry, Rigshospitalet, Copenhagen, Denmark.

## Conflict of interest

The authors declare that the research was conducted in the absence of any commercial or financial relationships that could be construed as a potential conflict of interest.

## Publisher’s note

All claims expressed in this article are solely those of the authors and do not necessarily represent those of their affiliated organizations, or those of the publisher, the editors and the reviewers. Any product that may be evaluated in this article, or claim that may be made by its manufacturer, is not guaranteed or endorsed by the publisher.
